# ‘Cry‐for‐help’ in contaminated soil: a dialogue among plants and soil microbiome to survive in hostile conditions

**DOI:** 10.1111/1462-2920.15647

**Published:** 2021-06-23

**Authors:** Eleonora Rolli, Lorenzo Vergani, Elisa Ghitti, Giovanni Patania, Francesca Mapelli, Sara Borin

**Affiliations:** ^1^ Department of Food, Environmental and Nutritional Sciences, DeFENS University of Milan, Via Celoria 2 Milan 20133 Italy

## Abstract

An open question in environmental ecology regards the mechanisms triggered by root chemistry to drive the assembly and functionality of a beneficial microbiome to rapidly adapt to stress conditions. This phenomenon, originally described in plant defence against pathogens and predators, is encompassed in the ‘cry‐for‐help’ hypothesis. Evidence suggests that this mechanism may be part of the adaptation strategy to ensure the holobiont fitness in polluted environments. Polychlorinated biphenyls (PCBs) were considered as model pollutants due to their toxicity, recalcitrance and poor phyto‐extraction potential, which lead to a plethora of phytotoxic effects and rise environmental safety concerns. Plants have inefficient detoxification processes to catabolize PCBs, even leading to by‐products with a higher toxicity. We propose that the ‘cry‐for‐help’ mechanism could drive the exudation‐mediated recruitment and sustainment of the microbial services for PCBs removal, exerted by an array of anaerobic and aerobic microbial degrading populations working in a complex metabolic network. Through this synergistic interaction, the holobiont copes with the soil contamination, releasing the plant from the pollutant stress by the ecological services provided by the boosted metabolism of PCBs microbial degraders. Improving knowledge of root chemistry under PCBs stress is, therefore, advocated to design rhizoremediation strategies based on plant microbiome engineering.

## The ‘cry‐for‐help’ hypothesis is poorly investigated in polluted environments

An impressive body of evidence proves that the root chemistry, that includes the metabolites exudated by the plant, their breakdown compounds and the soil microbe‐degraded sub‐products, plays a crucial role in shaping the structure and functionality of the root‐associated microbiome (Olanrewaju *et al*., 2019). The extent and nature of exudation varies with the age of the plant (Pausch and Kuzyakov, 2018), with the plant genetics (Cordovez *et al*., 2021), pathogen or symbiotic interaction or exposure to abiotic stresses, such as drought, high salinity, flooding, extreme temperatures or nutrient starvation (Vives‐Peris *et al*., 2020). The root chemistry drives the wellness of the holobiont, the meta‐organism resulting from the proficient interactions between the plant and the associated microbiome (Vandenkoornhuyse *et al*., 2015). In this win–win relationship, primary and secondary metabolites, volatile organic compounds, cells debris and by‐products of senescent root cells, accounting for ~10% of the total photosynthetically fixed carbon (Pausch and Kuzyakov, 2018), are allocated in the flux of root exudation to recruit, feed and influence the metabolism of specific microbial taxa (Liu *et al*., 2020). Recruited soil microbes establish in the rhizo/endosphere and assist the plant homeostasis by encoding for functionalities that extend the plant genome (Berendsen *et al*., 2012).

Despite the accumulating evidence, the molecular mechanisms that rule over the causal connection between (i) the plant phenotypes relevant for fitness, like development, growth, and health and (ii) the microbiome structure and functionality are still unknown.

In recent years, an experimental framework related mainly to pests‐ (Cotton *et al*., 2019; Liu *et al*., 2021) and herbivore‐attacked plants (Hu *et al*., 2018) has been validated to describe the proficient interplay between plant and microbiomes and that is encompassed in the ‘cry‐for‐help’ hypothesis (Rolfe *et al*., 2019). According to this theory, under phytopathogen or herbivore‐induced stress, the plant exudation pattern is shifted to release specific chemicals that favour the recruitment of beneficial microbes or of antagonists able to hinder the growth of pathogens (Rasmann and Turlings, 2016; Carrión *et al*., 2019; Rolfe *et al*., 2019). For example, *Fusarium oxysporum*‐infected cucumber roots were showed to increase tryptophan and decrease raffinose exudation, enhancing the colonization ability of the beneficial strain *Bacillus amyloliquefaciens* and counteracting the pathogen proliferation. On the other side, *B. amyloliquefaciens* releases tryptophan‐dependent auxin, in a positive feedback loop to support phytohormone homeostasis (Liu *et al*., 2017). The ‘cry‐for‐help’ hypothesis also explains the development of disease‐suppressive properties in infested soils (Wilkinson *et al*., 2019) and the maintenance of this legacy to subsequent plant generations growing in the same soil (Kong *et al*., 2019). For instance, through a metatranscriptomic approach targeting the sugar beet rhizosphere communities growing in a *Rhizoctonia*‐suppressive soil, bacteria affiliated to various families, (including *Oxalobacteraceae*, *Sphingobacteriaceae*, *Burkholderiaceae*, *Alcaligenaceae*, *Cystobacteraceae*, *Sphingomonadaceae*, *Cytophagaceae*, *Comamonadaceae* and *Verrucomicrobia*) were demonstrated to upregulate stress‐related genes to enhance survival strategies to cope with the pathogen (Chapelle *et al*., 2016; Expósito *et al*., 2017). Similarly, entomopathogenic nematodes are attracted by (E)‐ß‐caryophyllene, exudated by maize roots to hinder the damages caused by the insect *Diabrotica vergifera* (Rasmann *et al*., 2005).

In response to pathogen and pest attack, the plant microbiome is often enriched of bacteria that potentially can enhance plant defences or directly counteract the pathogen proliferation. Members of the genera *Chitinophaga*, *Chryseobacterium*, *Flavobacterium*, *Microbacterium*, *Pseudomonas*, *Sphingomonas*, *Stenotrophomonas* and *Xanthomonas* have been recently identified among the beneficial bacteria (Liu *et al*., 2020).

Beyond the ‘adapt or migrate’ strategy, plants could employ the ‘cry‐for‐help’ approach to benefit from microbial associations in contaminated environments, in case the pollutants are highly recalcitrant and toxic, poorly biodegradable and phyto‐extractable. Often plants fail to achieve full metabolism of persistent organic compounds, resulting in slow removal and incomplete degradation because they lack the catabolic enzymes necessary for their complete mineralization (Eapen *et al*., 2007; Schwitzguébel, 2017). As a consequence, xenobiotics commonly induce molecular injuries that disrupt biochemical, physiological and signalling processes and unbalance plant growth and survival (Ramel *et al*., 2012). The impact of xenobiotics exposure on rhizodeposition is underrated and the knowledge is sparse, limited to heavy metals like cadmium (Bali *et al*., 2020) and aluminium (Saha *et al*., 2020). The root system plunging in polluted soil, by changing the rhizodeposit fingerprint, resorts to the ‘cry‐for‐help’ to recruit microbes for their plant‐growth promoting activities, with the potential to release the plant from stress (Vergani *et al*., 2017a). Furthermore, the eventual enrichment of bacteria possessing the enzymatic machinery able to degrade the pollutant can decrease its local concentration, enhancing the detoxification in the root/rhizosphere compartments. This beneficial association is the keystone for rhizoremediation strategies, consisting in pollutant removal by microorganisms in the root zone (Balloi *et al*., 2010; Vergani *et al*., 2017b).

Classified within the most deleterious persistent organic compounds (POPs) for human, animal and ecosystem health (Stockholm Convention on POPs, 2015; Hens and Hens, 2017), polychlorinated biphenyls (PCBs) are a wide class of 209 congeners containing biphenyl with one up to 10 chlorine atoms. Although their production was banned worldwide in 1979 (Passatore *et al*., 2014), PCBs still represent a threat for their teratogenic (Berghuis and Roze, 2019), carcinogenic (Magoni *et al*., 2019; Guo *et al*., 2020), mutagenic potential (Murati *et al*., 2020), together with their recalcitrance in the environment (Simhadri *et al*., 2020), persistence and biomagnification in the food web (Amutova *et al*., 2021). For land reclamation, PCB‐polluted soils should be excavated for off‐site treatment by solvent extraction, thermal alkaline dechlorination, incineration or landfilling (Campanella *et al*., 2002). While these techniques are expensive and, in many cases, almost infeasible due to the general large extension of the contamination (Van Aken *et al*., 2010), rhizoremediation offers a sustainable, potentially efficient and cost‐effective technology for PCBs removal from soil (Vergani *et al*., 2017b).

In this review, the role of root exudation as plant driver of the ‘cry‐for‐help’ strategy in PCBs contaminated soil is discussed, showing its central role in recruiting and sustaining the microbial services to reduce the contaminant load, thus restoring the holobiont health.

## Plant fitness falters in PCBs contaminated soil

The routes of PCBs uptake and translocation in plant tissues are largely unknown: as foreign molecules not naturally present in the environment, they presumably enter plant tissues by hijacking the pathways required for the uptake of soil nutrients (Greenwood *et al*., 2011).

**Fig 1:**
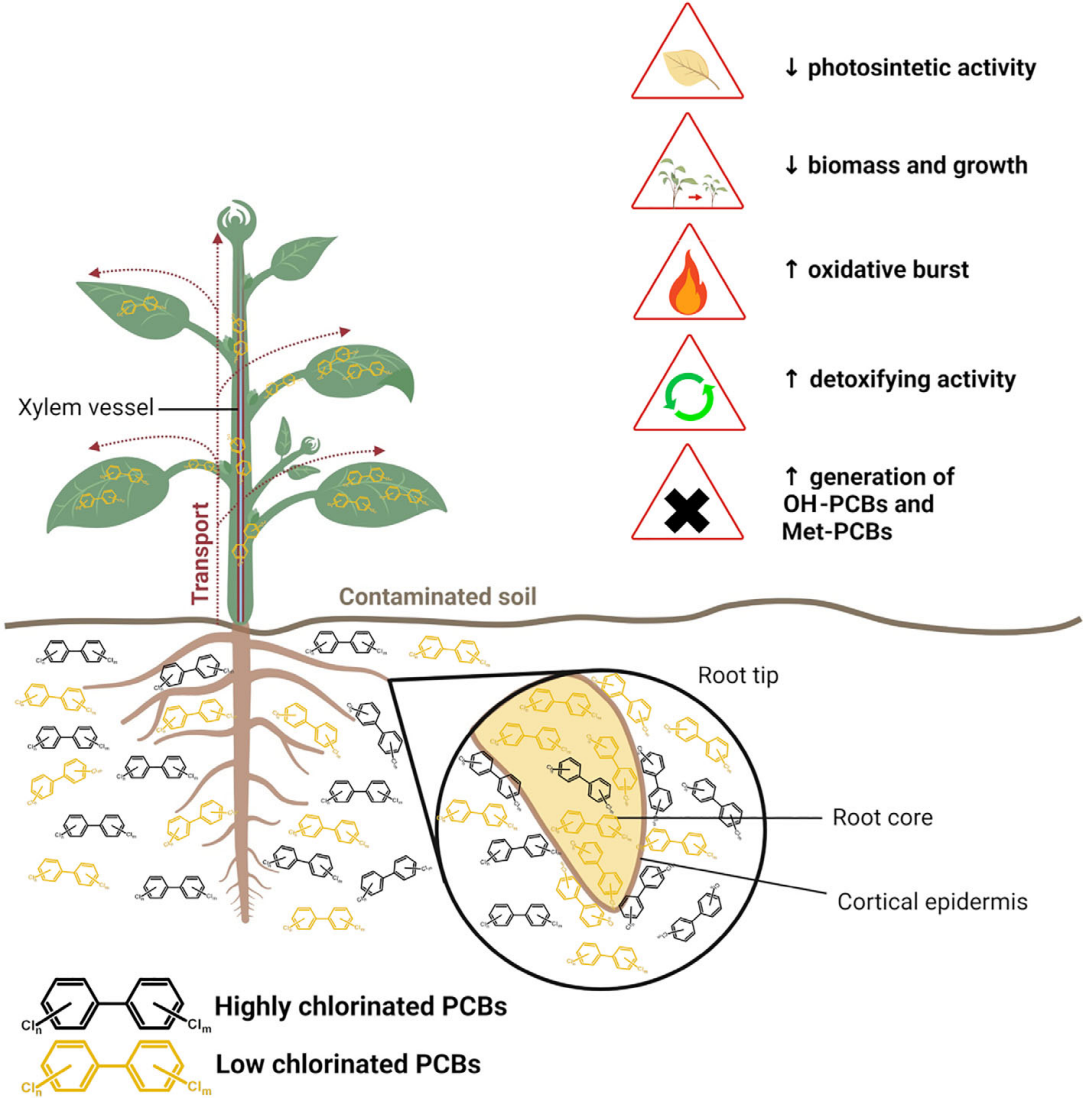
Polychlorinated biphenyls (PCBs) distribution in plant tissues and phytotoxic effects. Several studies reported a differential distribution of low and highly chlorinated PCBs in plant tissues. The external cortical epidermis of the root can interact and be enwrapped by PCBs with a different degree of chlorination, but low chlorinated PCBs are favoured in entrance in the root internal core and then can be translocated through the xylem sup in the plant aerial compartments, both stem, shoot and leaves. Plants are unable to fully mineralize PCBs and the exposition to these toxic pollutants result in a plethora of phytotoxic effects, affecting plant growth and health.

PCBs mainly absorb on root epidermal surfaces and then diffuse in less amount within the root tissues (Fig. 1), as observed in alfalfa plants exposed to PCB 28 (Teng *et al*., 2017). The root capacity to absorb extremely hydrophobic compounds like PCBs depends on the thickness of waxes on the root epidermis (Zhang *et al*., 2009). Pectins, as well, may mediate PCBs accumulation in the root tissues: the Arabidopsis mutant *quasimodo1*, affected in a galacturonosyltransferase enzyme, showed a reduced amount of PCB 18 in the root system (Bao *et al*., 2013). Although pectins are well documented in chelating heavy metals (Shao *et al*., 2021), their role in PCBs phyto‐extraction remains elusive.

Several lines of evidence suggest that lower chlorinated PCBs are preferentially uptaken and accumulated by the roots than higher chlorinated PCBs (Asai *et al*., 2002; Luo *et al*., 2020; Fig. 1). Once collected in the root tissue, PCBs can be then translocated at some extent in the epigeal compartment (stem and leaves), as observed in pumpkin plants cultivated in a soil polluted by the commercial PCBs mixture Aroclor (Whitfield Åslund *et al*., 2008). Similar indications were observed in poplar plants grown in a hydroponic medium artificially contaminated by PCBs where the higher chlorinated compounds were compartmentalized in the root and the lower chlorinated ones were mobilized in the stem (Liu and Schnoor, 2009). The translocation seems to occur through the xylem sup, as documented in pumpkin plants (Greenwood *et al*., 2011) (Fig. 1). PCBs concentration decreases in the sup along the shoot as the distance from the roots increases, with low chlorinated compounds that are more easily mobilized toward the plant aerial compartments (Whitfield Åslund *et al*., 2007; Greenwood *et al*., 2011). PCBs translocation seems to be influenced not only by the PCB congener and the chlorine substitution pattern, but also by the plant species (Asai *et al*., 2002; Iwabuchi *et al*., 2020).

Controversial conclusions were drawn for PCBs‐induced phytotoxicity, which has been estimated on a wide array of heterogeneous experimental settings, including *in vitro* assay or field trial with different plant species at various developmental stages and different PCB congener's mixtures (Zeeb *et al*., 2006; Wang *et al*., 2017). Thus, variable plant responses to PCBs exposition were recorded. Some studies reported a phytotoxic effect given by PCBs on plant growth only at high concentrations (Jin *et al*., 2011; Wang *et al*., 2017), and low to moderate negative effect for lower levels of PCBs contamination (Zeeb *et al*., 2006; Deavers *et al*., 2010;Subramanian *et al*., 2018; Urbaniak *et al*., 2020). On the contrary, in some cases PCB were described to induce plant growth stimulation (Subramanian *et al*., 2018; Urbaniak *et al*., 2020), claiming for a hormesis effect (Calabrese, 2014).

A possible explanation to the observed variability in PCBs toxic effect is linked to the above‐mentioned factors affecting PCB uptake by the roots and translocation, such as the plant species and PCB hydrophobicity and bioavailability, strongly dependent on the congener and substitution patterns (Van Aken *et al*., 2010).

Xenobiotics responses in higher plants are still cryptic, although it is ascertained that POPs could disrupt molecular and biochemical processes, thus affecting signalling circuits (Ramel *et al*., 2012). Transcriptomics study of *Arabidopsis* plants exposed to 2,5‐dichlorobiphenyl (Jin *et al*., 2011) and tetrachlorobiphenyl (Subramanian *et al*., 2018) showed an enrichment in gene functions related to the metabolism of toxic substances and to response to oxidative stress, oomycetes and bacteria, confirming, as observed for other stresses, the convergence of biotic and abiotic signalling responses (Fujita *et al*., 2006).

Phenotypically, PCBs exposure showed to cause fall in germination rate (Meier *et al*., 1997; Subramanian *et al*., 2018), reduction of the plant biomass (Kučerová *et al*., 2000), decrease in the chlorophyll content with leaf bleaching effect (Bao *et al*., 2013) and burst of oxidative stress (Ahammed *et al*., 2013) (Fig. 1).

To cope with xenobiotics, plants adopt a three‐steps detoxifying strategy, summarized in the green liver model (Sandermann, 1994), that include: (i) activation of POPs by oxidation to produce hydroxylated molecules with a higher solubility; (ii) conjugation to generate adducts with glutathione to decrease the POPs toxicity and (iii) sequestration of the conjugates in the vacuole or incorporation into plant cell wall (Coleman *et al*., 1997).

Unfortunately, this detoxification mechanism is inefficient and has a limited impact on plant release from PCBs‐induced stress (Schäffner *et al*., 2002). Indeed, many evidence suggest that plant catabolism of PCBs is limited to tetrachlorinated and lower congeners (Kučerová *et al*., 2000; Van Aken *et al*., 2010), and, moreover, the substitution patterns of these molecules can dramatically affect their recalcitrance (Rezek *et al*., 2007; Van Aken *et al*., 2010).

Furthermore, the initial metabolism of PCBs in plants involves the activity of monooxygenases of the cytochrome P‐450 family and peroxidases that generate hydroxylated (OH‐) and methoxylated (MeO‐) PCBs as observed in rice, maize, wheat and *Arabidopsis*, having an enhanced toxicity than the parental molecule (Sun *et al*., 2016; Subramanian *et al*., 2018; Sun *et al*., 2018; Lin *et al*., 2020), posing a further stress for plant physiology and an environmental risk for human and ecosystem health (Wu *et al*., 2014). Exposure to OH‐ and MeO‐PCBs in rice triggers an enhanced energy demand, promoting the catabolism of pyruvate, the TCA cycle (tricarboxylic acid), the transfer of acetyl groups into mitochondria and finally resulting in a higher biomass reduction than the parental 2,3,4,5‐tetrachlorobiphenyl treatment (Lin *et al*., 2020). The plant displays the most efficient detoxification effect, glutathione‐mediated, only after exposure to the parental congener, while a vast array of less effective antioxidant mechanisms are activated by OH‐ and MeO‐PCBs, explaining, therefore, their higher phytotoxicity (Lin *et al*., 2020).

Recently, in the soil of a historically polluted site in Italy (Di Guardo *et al*., 2017), sulfonated‐ and hydroxy‐sulfonated‐PCBs were detected (Bagnati *et al*., 2019). The synthesis of these sulfonated compounds, maybe through the action of the glutathione S‐transferase enzyme (Blanchette and Singh, 2003), has been hypothesized to be part of the detoxifying strategy to enhance the PCBs mobility and bioaccessibility (Bagnati *et al*., 2019) as already demonstrated for other molecules potentially harmful for the plant (Chen *et al*., 2015). Plant and environmental fate of such modified PCBs remains still to be elucidated.

Knowledge about plant metabolism of xenobiotics is still fragmentary, although it was described that plants contribute to de‐adsorb PCBs from soil particles and mobilize them, up to 62% of the initial PCB contamination (Chekol *et al*., 2004). During growth and development, plants influence in the soil surrounding root the abundance and composition of dissolved and particulate organic carbon (DOC and POC respectively) that are the main drivers for the chemical movement of hydrophobic compounds in soil (Terzaghi *et al*., 2020). The increased PCB bioavailability could further compromise plant growth. Therefore, the selection of plant species effective for PCB phytoremediation like *Medicago sativa* (alfalfa), *Lespedeza cuneate* (Chinese bushclover), *Lathyrus sylvestris* (everlasting pea), *Phalaris arundinacea* (reed canary grass), *Cucurbitaceae* (cucurbits), *Sparganium* (bur‐reed), *Salix alaxensis* (Alaska willow) and *Picea glauca* (white spruce; Chekol *et al*., 2004; Ficko *et al*., 2010; Slater *et al*., 2012; Terzaghi *et al*., 2020), have been suggested as crucial for land reclamation of PCB‐polluted areas. The increase of PCB bioavailability in the rhizosphere makes these highly recalcitrant molecules more accessible also for microbes featuring the enzymatic arsenal to degrade them (Vergani *et al*., 2017b), generating propitiatory conditions for the ‘cry‐for‐help’ strategy. Although it can be supposed that PCB‐ induced effect on plant physiology and stress response could affect rhizodeposition, the changes in the root chemistry have still to be mechanistically demonstrated.

## PCBs‐degradative potential of soil microbiomes

Despite PCBs have been introduced in the environment by human activity in relatively recent times, bacterial communities inhabiting polluted soils are well known for their potential to degrade this class of molecules, via both dechlorination and cleavage of the biphenyl ring. These capacities are probably derived from the cycling of natural organohalogens (Atashgahi *et al*., 2018; Temme *et al*., 2019) and the degradation of plant‐derived aromatic organic compounds (Fuchs *et al*., 2011) as a consequence of structural similarity.

PCB dechlorination has been confirmed only as anaerobic reductive activity among members of the phylum Chloroflexi, including *Dehalococcoides*, *Dehalogenimonas* and *Dehalobium*, either by organohalide‐respiration or by co‐metabolism (Xiang *et al*., 2020). There are evidences that also bacteria classified as *Clostridia* (Firmicutes) and *Geobacteraceae* (δ‐Proteobacteria) are involved in this activity (Wang and He, 2013; Praveckova *et al*., 2016).

The most studied PCB‐dechlorinating bacteria are strains belonging to the only described species of the genus *Dehalococcoides, D. mccartyi*. This species is an obligate organohalide‐respiring bacteria (OHRB), strictly anaerobic, with a small‐sized genome yet encoding for several reductive dehalogenase enzymes (RDases) active towards a broad range of chlorinated compounds that represent its sole source of energy (Taş *et al*., 2010; Bedard, 2014). *Dehalococcoides* presence and activity was reported in different environments such as polluted soils, sediments and groundwater (Taş *et al*., 2010), however, RDases responsible for PCB dechlorination were described for the first time in 2014 (Wang *et al*., 2014). Combining cultural techniques with metagenomic and metatranscriptomics, the authors identified in three new isolates of *D. mccartyi* different reductive dechlorinases, PcbA1, PcbA4 and PcbA5, catalysing the *meta* and *para‐*dechlorination of several PCB congeners of the mixture Aroclor 1260 (Bedard, 2014).

The role of biotic and abiotic factors influencing the activity of OHRB in soils is still poorly understood; however, the interactions with other bacteria populations, the soil type and water content seem to be crucial. A pivotal role is likely played by other bacteria providing electron donors, carbon sources and the essential corrinoid cofactors for RDases (Men *et al*., 2012). It has been proposed that *D. mccartyi* forms a core community with *Methanosarcina* and *Desulfovibrio* populations, which have been observed to support PCB dechlorination mediating acetate and hydrogen sources (Wang *et al*., 2019). The structure and activity of organohalide‐degrading communities are also connected to soil edaphic factors and the presence of anaerobic niches allowing the metabolism of strictly anaerobic OHRB. In a recent study, the soil water content was identified as the main parameter positively related to PCB dechlorination, increasing the cell‐to‐contaminant availability (Shen *et al*., 2021). Both the presence of oxygen‐depleted niches, due to root respiration activity, and the increase in water retention due to the release of exopolysaccharides, indicates the plant rhizosphere as a potential hot spot for PCB reductive dechlorination.

As most of the knowledge about PCB dechlorination deals with OHRB, far less is known about the possible role of other bacteria and associated non‐reductive enzymes. According to recent metagenomic studies, both respiratory and non‐respiratory processes seem to be widespread in terrestrial ecosystems and involved in chlorinated‐natural organic matter (Cl‐NOM) cycling (Weigold *et al*., 2016; Temme *et al*., 2019). RDases and hydrolytic or oxidative dehalogenase enzymes were retrieved in many environments, including sediments from urban lakes and in enrichment cultures from a PCB‐polluted soil, and the abundance of these enzymes increased in the presence of Cl‐NOM (Temme *et al*., 2019). These findings suggest that both respiring and non‐respiring dehalogenating microbial communities may be important for the dechlorination of PCB in contaminated soils, though this hypothesis requires to be further investigated.

The possibility of aerobic degradation of PCB is tightly related to the number and position of chlorine substitutions. Indeed, highly chlorinated congeners are not accessible to aromatic ring‐degrading enzymes and therefore their metabolism is dependent on the removal of chlorine by dehalogenating microorganisms (Borja *et al*., 2005). PCB aerobic catabolism by soil bacteria has been extensively reviewed (Abraham *et al*., 2002; Field and Sierra‐Alvarez, 2008; Furukawa and Fujihara, 2008). The process is known to occur mainly through the enzymes of the biphenyl pathway, encoded by the *bph* operon that is shared among different bacterial phyla, probably as a result of the ancient evolution of the degradation of biphenyl‐related molecules of plant origin such as the lignin complex (Fuchs *et al*., 2011). These genes are often found on mobile genetic elements and therefore may be spread through horizontal gene transfer. Recent studies showed that *bph* clusters of β‐ and γ‐proteobacteria were located as integrative conjugative elements both on plasmids or on the chromosome and could be transferred among bacterial cells (Suenaga *et al*., 2017; Hirose *et al*., 2019).

The aerobic biphenyl degradation pathways potentially lead to the conversion of the biphenyl ring into benzoate (upper pathway) and to further degradation into the TCA cycle (lower pathway). The key enzyme responsible for initiating PCB degradation is biphenyl dioxygenase (BphA), a multi‐component Rieske non‐heme iron oxygenase encoded by *bphA1A2A3A4*. Differences in *bphA1* sequence, encoding for BphA α‐subunit, are determinant for substrate specificity and consequently biodegradation capability of different PCB congeners, as found among the most studied PCB‐degrading strains such as *Pseudomonas furukawaii* KF707, *Paraburkholderia xenovorans* LB400 and *Rhodococcus jostii* RHA1 (Vergani *et al*., 2017a). Other *bph* operons of bacteria belonging to different species have been sequenced, presenting some differences in their genetic organization and regulation, as well as different enzymes specificity towards *ortho‐, meta‐* or *para‐* substituted congeners. However, the characterization of the degradation pathways confirmed that their function is highly conserved (Gómez‐Gil *et al*., 2007; Ridl *et al*., 2018; Garrido‐Sanz *et al*., 2020).

Although *bph* pathways associated to diverse strains are incomplete, causing the accumulation of intermediates that can be toxic for other bacteria and organisms, it is important to note that the biodegradation processes by soil bacterial communities likely occur through a complex metabolic network, rather than a linear series of reactions, and involve the interaction of different populations of degraders active at different catabolic steps (Leewis *et al*., 2016; Duarte *et al*., 2017). A few studies coupling stable isotope probing (SIP) with 16S rRNA gene sequencing showed that biodegradation potential in PCBs polluted soils is widespread among different species, with a prevalence of Actinobacteria and Proteobacteria, suggesting that active populations vary depending on site and environmental conditions (Uhlik *et al*., 2013; Leewis *et al*., 2016; Jiang *et al*., 2018). Also, aromatic ring‐hydroxylating dioxygenases metagenomic studies highlighted a high degree of sequence diversity, indicating that the catabolic potential hosted by microbial communities inhabiting contaminated soils remains widely unexplored (Aguirre De Cárcer *et al*., 2007; Iwai *et al*., 2010; Standfuß‐Gabisch *et al*., 2012). In a recent study, the metabolic pathways involved in biphenyl biodegradation by a bacterial consortium isolated from PCBs polluted soil were identified via metagenomic analysis, revealing three different pathways converting biphenyl into benzoate and five pathways degrading benzoate to the TCA cycle intermediates (Garrido‐Sanz *et al*., 2018).

This approach also allowed to reveal which bacterial populations carried out specific reactions in the network, showing that the *bphABCD* operons of *Rhodococcus* strains were responsible of all the three upper pathways. The lower pathways were mainly initiated by *Pseudomonas* and *Bordetella* via benzoate 1,2‐dioxygenase (*benABC*), by *Variovorax* and *Achromobacter* that were involved in benzoate ligation with acetyl‐CoA (benzoate CoA‐ligase, *blcA*), while 4‐hydroxybenzoate 3‐monooxygenase (*PobA*) were associated to *Pseudomonas*, *Bordetella*, *Achromobacter*, *Ralstonia* and *Rhodococcus*. A more complex bacterial community was then involved in the subsequent catabolism of intermediates.

Besides bacteria, PCB degradation has been focused mainly on wood‐decay fungi, which have been studied due to their ligninolytic activity. In fact, as xylophagous organisms, they produce extracellular non‐specific laccases and peroxidases enzymes that efficiently attack the biphenyl ring of several PCBs congeners. Intracellular enzymes such as cytochrome P‐450 and dehydrogenases are also involved in the detoxification process (Čvančarová *et al*., 2012; Stella *et al*., 2017). Instead, the degrading activity of soil‐dwelling fungi is poorly studied. Some species of filamentous fungi belonging to the genus *Penicillium, Aspergillus, Scedosporium, Doratomyces, Myceliophthora, Phoma* and *Thermoascus* were isolated from PCBs polluted soils, and their degradation potential was characterized in a few studies (Tigini *et al*., 2009; Mouhamadou *et al*., 2013; Germain *et al*., 2021). PCB depletion is thought to occur mainly through the activity of laccases, however the biodegradation pathways of these microorganisms need further investigation.

## Plant–microbes dialogue mediated by the root exudates: sources of carbon, co‐metabolites or signal molecules for PCB degrading bacteria

**Fig 2:**
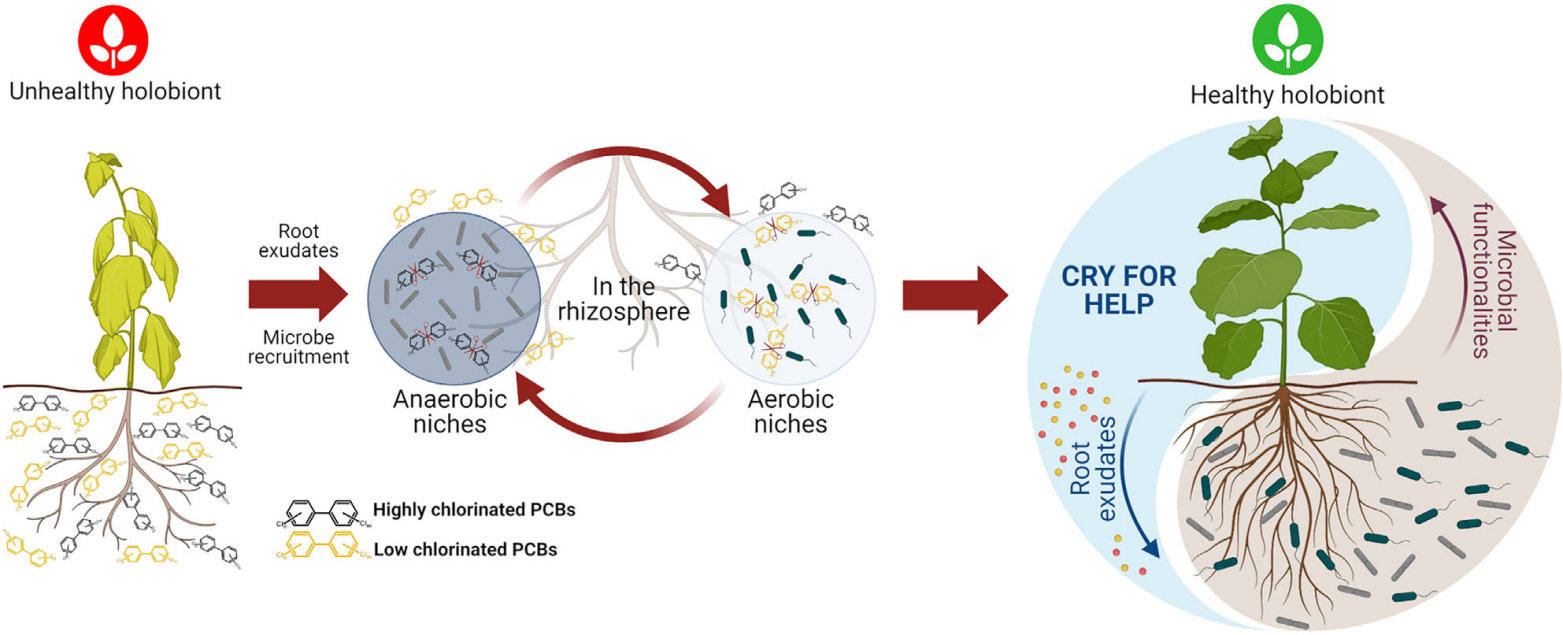
The complex network interaction between the plant ‘cry‐for‐help’ strategy and the ecological services provided by the degrader microbiome sustain the holobiont fitness in polychlorinated biphenyls (PCBs) contaminated soils. The PCB‐induced phytotoxic effects negatively impact the growth and health of plants growing in polluted soils, which modify their root chemistry in a ‘cry‐for‐help’ strategy to recruit, feed and sustain PCB‐degrading microbes in the rhizosphere. Plant primary and secondary metabolites are valuable candidates to support the microbial aerobic co‐metabolism and anaerobic dechlorination of PCBs. Different arrays of microbial populations are involved in PCBs degradation, acting in a metabolic network and in different aerobic and anaerobic micro‐niches that establish in the rhizosphere also as consequence of microbial growth and PCB metabolism, favouring further clean‐up of the pollutant. The ‘cry‐for‐help’ approach and the microbial functionalities encoded by the recruited microbiome contribute to foster this healthy system, restoring the holobiont fitness.

Prolonged exposure to xenobiotics in historically polluted areas promotes a Darwinian selection of holobionts that adapt as a whole to thrive in presence of the contaminant (Osmanovic *et al*., 2018). The ‘cry‐for‐help’ contributes to the structure of the holobiont, driving the ecosystem services provided by the microbial functionalities involved in organic pollutants degradation (Uhlik *et al*., 2013) (Fig. 2). Furthermore, plants adopt a variety of strategies to facilitate the microbial viability and metabolism in an adverse environment, challenged by the high organic contaminant concentration (Di Guardo *et al*., 2017). Such adverse conditions could lead to the entry of bacterial cells in a dormant phase, like for example the so‐called ‘viable but non culturable’ (VBNC) state, from which they can resuscitate under proper environmental and nutritional stimuli (Murugan and Vasudevan, 2018). PCBs pose a threat to bacterial cells viability, as they may accumulate in the cytoplasmatic membrane and disrupt its functionality (Chávez *et al*., 2006). Notably, PCB toxicity, as for plants, even for bacteria is mostly degradation‐dependent, with catabolic products such as dihydrodiols, dihydroxybiphenyls and catechols affecting cell growth and viability much more than the parental compounds due to their hydrophilic substituents, which increase the impact on the cell membrane structure and function (Cámara *et al*., 2004). To cope with these effects, the strong degrader *Paraburkholderia xenovorans* LB400 has been shown to activate an efficient detoxification response that is induced only upon PCB degradation (Parnell *et al*., 2006). Besides the activation of such detoxification pathways, bacterial tolerance and stress response to PCBs and related metabolites is variable and includes also structural adaptive changes of the cell membrane and the expression of nonspecific stress shock proteins (Kim and Masunaga, 2005; Chávez *et al*., 2006).

Among the vast array of mechanisms that plants adopt to set off the ‘cry‐for‐help’, the release of primary metabolites like sugars, amino acids and organic acids can support the growth of specific microbial taxa (Macek *et al*., 2000). The release of a specific profile of root exudates had been considered responsible of the decline in PCBs concentration in *Salix caprea* and *Armoracia rusticana* vegetated soil correlated positively with the population size of culturable PCB degraders enumerated in the rhizosphere of the two plant species (Ionescu *et al*., 2009). Moreover, the root‐associated soil was enriched with bacteria showing a broad PCB congeners catabolism, belonging to the *Pseudomonas*, *Burkholderia* and *Sphingobacterium* genera (Ionescu *et al*., 2009).

Besides being used as nutrients, plant primary metabolites can be deployed as electron donors to support aerobic co‐metabolism or anaerobic dechlorination of PCBs (Leigh *et al*., 2006). As a side effect, the enhanced aerobic metabolism consumes oxygen, favouring the formation of anaerobic niches for dehalogenation of high‐chlorinated PCBs (Chaudhry *et al*., 2005). This will presumably generate a positive feedback mechanism. The decrease of PCB concentration in the rhizosphere would remove the inhibitory effect of these molecules on plant growth, favouring root development and penetration in soil. As a consequence, oxygenation of soil particles would increase, driving the aerobic oxygenases‐mediated degradation of PCBs and generating a cascade effect of oxygen consumption with important implications for the further anaerobic depletion processes (Fig. 2).

Besides primary metabolites, plants can contribute in accelerating microbial PCB degradation by releasing secondary metabolites that act as inducers or co‐metabolites and trigger the expression of the microbial enzymatic degradation machinery (Musilova *et al*., 2016). These root exudate compounds include phenolic molecules like flavonoids, terpenoids, steroids and alkaloids that often share a high structure similarity with PCBs (Jha *et al*., 2015). Flavonoids, in particular, encompass a huge variety of molecules that influence plant development, response to stress and interaction between plants, microbes and animals (Mathesius, 2018) and have been implicated in the plant‐mediated induction of PCB‐degrader mechanisms in soil microorganisms (Toussaint *et al*., 2012; Pham *et al*., 2015; Subramanian *et al*., 2018). An altered exudation profile of flavonoids in *Arabidopsis* mutants affected the colonization and consequently the PCB degradation ability of *Pseudomonas putida* PLM2 (Narasimhan *et al*., 2003). Indeed, flavone and isoflavone were demonstrated to act as inducers of the biphenyl degradative pathway in *Rhodococcus erythropolis*, providing the energy necessary for the contaminant metabolism (Toussaint *et al*., 2012; Pham *et al*., 2015).

Terpenes, composed of two or more isoprene units, are responsible for the fragrances of fruits and essential oils and act as chemoattractants for soil microbes (Huang and Osbourn, 2019). The PCB degrader *Pseudomonas stutzeri*, exposed to the terpenes limonene and carvone, showed differential degradation ability towards the PCB mixture Delor 103, that was dependent on the concentration of the inducer (Tandlich *et al*., 2001).

The role of primary and secondary metabolites can also overlap by acting as attractants to stimulate chemotaxis toward the rhizosphere, as demonstrated for the strain *Rhodococcus erythropolis* U23A that showed motility toward phenolic compounds exudated by *Arabidopsis* roots (Toussaint *et al*., 2012). Furthermore, variation in the concentration profile of exudated nutrients would affect the residency in the rhizosphere of microbial taxa characterized by diverse life‐history strategies (i.e. copiotrophs vs. oligotrophs). This effect was recently documented by a metabolomic approach on the exudates released by *Avena barbata*. During plant development, besides the fast‐growing taxa that rapidly consumed the labile exudates, there were a high proportion of slow‐growing strains adapted to specialized metabolic niches (Zhalnina *et al*., 2018).

Rhizodeposition can also occur through decomposition of deposited litter and lysis of sloughed‐off root cells (Dennis *et al*., 2010), presumably containing a diverse array of metabolites. Past (Hernandez *et al*., 1997; Musilova *et al*., 2016) and recent studies (Terzaghi *et al*., 2019; Terzaghi *et al*., 2021) pinpoint for the effectiveness of the combination of compost supply and plant exudation in increasing cell viability and the microbial activity of PCB‐degrading microbiome, while decreasing the load of higher and lower‐chlorinated PCBs (Hayat *et al*., 2019; Di Lenola *et al*., 2020).

Through the mixture of organic compounds released by roots, litter decomposition and sludged root cells, plants could contribute to resuscitate microbes from the dormant VBNC state, in which they can enter as an adaptive response limiting their growth and division (Murugan and Vasudevan, 2018). Plants contribute, moreover, to increase PCB mobility and bioavailability for microbial degradation through the release of molecules that can act as surfactants (Campanella *et al*., 2002) or affecting the composition of particulate organic carbon in soil (Terzaghi *et al*., 2020).

Recent evidence suggests that the ‘cry‐for‐help’ is not a merely unidirectional strategy employed by the plant to maximize its fitness through the services provided by beneficial microbes. It implies multiple interactions and a molecular dialogue of the holobiont components, the plant and its microbiome, aimed to preserve its functionality under adverse environmental conditions (Fig. 2). Once established in the rhizosphere, after recruitment and feeding through root exudation, microbes can manipulate rhizodeposition to ensure and consolidate their metabolic niche (Korenblum *et al*., 2020). For example, the release of coumarins, like scopoletin, by *Arabidopsis* roots under iron starvation, showed to improve the colonization by the beneficial strain *P. simiae* that, in turn, stimulated the further exudation of scopoletin to counteract the growth of fungal and bacterial competitors that are sensitive to the antimicrobial activity of coumarins (Stringlis *et al*., 2018).

The chemistry and mechanisms of communication between the plant host and PCB‐degrading soil microorganisms open new challenges for ecological investigation.

## Concluding remarks

Today, an arsenal of new methodologies is available to unveil the complex crosstalk between plants and the associated microbiome (Park and Ryu, 2021). The use of *Arabidopsis* mutant lines (Huang and Osbourn, 2019; Voges *et al*., 2019) and the *‐omics* approaches, including culturomics for setting up microbial synthetic communities (Ziegler *et al*., 2013; de Souza *et al*., 2020) and metabolomics to identify the plant exudate compounds (Jaini *et al*., 2017; Pétriacq *et al*., 2017; Kawasaki *et al*., 2018; Dietz *et al*., 2020) potentially will allow to decipher the messages involved in the ‘cry‐for‐help’ dialogue adopted by plants as response strategy to cope with different environmental stresses (Liu *et al*., 2020). As recently highlighted by Stringlis and colleagues (2018), who investigated the plant response to iron deficit condition, the microbiome is not merely a receiving component of this dual system. On the contrary, the ability of certain microbes to promote the synthesis and root release of specific compounds ultimately influences the composition of the microbiome itself. The bacteria‐mediated induction of specific plant genes recalls the fine talk taking place in the establishment of the legume‐*Rhizobium* symbiotic relationship, where the bacterium, after rhizoplane colonization, causes morphological changes in the root epidermis related to the expression of the plant early‐noduling genes and, from the other side, in response to the flavonoids exudated by roots initiate the synthesis of the Nod Factors which in turn act as transcriptional factors for the plant (Geurts and Bisseling, 2002).

A co‐adaptative strategy is established between recruited microorganisms and plants exposed to a particular stress, as observed for drought (Williams and de Vries, 2020), metal toxicity (Timm *et al*., 2018), plant predation (Adaikpoh *et al*., 2020) and nutrient limited growth conditions (Ham *et al*., 2018) to support plant growth under these specific abiotic stressors (Liu *et al*., 2020). The coumarin scopoletin breaks this paradigm, showing that a molecule exudated under iron starvation is also involved in counteracting pathogen proliferation and favouring the recruitment of beneficial microbes (Stringlis *et al*., 2019). Identifying similarities and peculiarities in the root exudation profile responding to different abiotic stresses will contribute to decipher the mechanistic aspects of microbial communities assembly upon the plant ‘cry‐for‐help’ strategy, and the study of root exudation in contaminated soils will highlight the plant‐microorganisms interplay in pollutant degradation.

Improving the knowledge on plant secondary metabolites and root exudates effect in response to PCBs polluted soil paves the way for microbiome manipulation to gain ecological services like rhizoremediation. Though most of the ‘cry‐for‐help’ pioneering studies have been realized using the model plant *Arabidopsis thaliana*, future research could be addressed on plant species that demonstrated to induce a decrease in PCBs concentration by biostimulating the microbiome of historically and highly polluted soils (e.g., *Festuca arundinacea*, *Medicago sativa*, *Cucurbita pepo ssp. pepo*, Terzaghi *et al*., 2019). A fine metabolomic characterization of the exudation pattern of these plant species challenged in PCBs contaminated soil, coupled with metagenomic studies aimed at identifying the soil microbiome response in terms of functional traits related to biodegradation, will thus represent a milestone to steer the recruitment of PCB‐degrading microbial populations and design effective rhizoremediation strategy based on microbiome engineering.
